# Rapid‐Forming Conductive Antifouling Hydrogel Coating Enables Magnetically Guided Electrochemical POCT for Direct at‑Home Blood Testing

**DOI:** 10.1002/advs.77696

**Published:** 2026-09-16

**Authors:** Menghan Li, Kai Su, Ruiwei Hu, Ke Gao, Yuanjie Liu, Lu Zhang, Ningke Fan, Yirong Chen, Wei Cheng, Shijia Ding, Haiping Wu

**Affiliations:** ^1^ The Center for Clinical Molecular Medical Detection The First Affiliated Hospital of Chongqing Medical University Chongqing People's Republic of China; ^2^ College of Laboratory Medicine , Key Laboratory of Clinical Laboratory Diagnostics (Ministry of Education) Chongqing Medical University Chongqing People's Republic of China; ^3^ Department of Clinical Laboratory Qianjiang Key Laboratory of Chongqing Qianjiang Central Hospital Laboratory medicine Chongqing University Qianjiang Hospital (Qianjiang Central Hospital of Chongqing) Chongqing People's Republic of China; ^4^ Sichuan‐Chongqing Joint Key Laboratory for Pathology and Laboratory Medicine Jinfeng Laboratory Chongqing People's Republic of China

**Keywords:** anti‐fouling hydrogel coating, at‐home testing, electrochemical POCT, immunoassay, magnetic‐guided enrichment

## Abstract

Electrochemical point‐of‐care testing for complex clinical samples is frequently hampered by matrix interference, signal instability, and laborious electrode modification procedures. Herein, we report a magnetic portable electrochemical immunosensor utilizing a conductive, anti‐fouling hydrogel coating composed of sodium alginate, polyethylene glycol, and graphene (SPG). Upon calcium‐ion triggering, the SPG solution forms a stable electrode hydrogel coating within 1 min, effectively resisting nonspecific adsorption while maintaining low‑impedance electron transfer. Guided by a 3D‐printed magnetic apparatus, the MpECis directly captures target‐bound magnetic complexes, enabling rapid enrichment and electrical readout within 25 min. By implementing time‑domain averaging over the enzymatic reaction plateau, the coefficient of variation of electrical signals generated by nanoenzyme catalysis was reduced to 3.4%, markedly improving measurement reproducibility. The clinical practicality of the SPG MpECis platform was validated through three independent cohorts, including detection of cardiac troponin I in 59 serum samples, quantification of extracellular vesicles in 47 plasma samples, and detection of C‐reactive protein in 17 fingertip blood samples. Compared with the reference method, the area under the curve of this method are 0.99, 0.93, and 1.00, respectively. This platform offers a practical route for stable electrochemical diagnostics in complex biological matrices and home‐based precision medicine.

## Graphical abstract

The SPG‐MpECis electrochemical POCT platform developed in this study enables direct detection of biomarkers in complex biological samples such as serum and fingerstick blood, demonstrating high concordance with results from clinical reference methods and holding broad potential for at‐home testing applications.

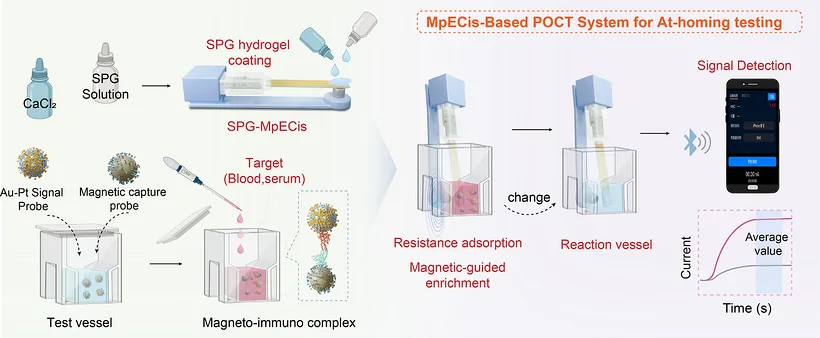

## 1 Introduction

As the global population ages and the demand for comprehensive management of chronic diseases, including cardiovascular disorders and cancer, continues to rise, decentralized, high‐frequency health monitoring at home is gaining growing recognition as an essential complement to conventional healthcare systems [1, 2]. Conventional centralized testing paradigms, however, are ill‐equipped to meet the urgent patient need for real‐time, accessible, and cost‐effective diagnostics. This gap has spurred the rapid emergence and evolution of point‑of‑care testing (POCT) technologies suitable for primary care settings and even residential use [3, 4, 5, 6, 7, 8, 9, 10, 11, 12]. Among the diverse POCT modalities, lateral flow immunoassays have achieved notable popularity owing to their operational simplicity and low cost [13, 14, 15, 16]. Nevertheless, their intrinsic drawbacks, including semi‐quantitative or merely qualitative readouts, limited sensitivity, and reliance on colorimetric or fluorescent signals that are susceptible to ambient light interference, hinder their ability to satisfy the growing demand for precise quantitative biomarker detection in complex physiological matrices [17, 18, 19, 20].

The distinctive performance advantages of electrochemical POCT technology have opened up a novel avenue for accurate at‑home testing. At its core, this approach relies on the direct or indirect transduction of biological events into quantifiable electrical signals. Such a transduction mechanism not only enables high‐sensitivity quantitative analysis but also facilitates seamless integration with miniaturized electronic devices and offers straightforward, cost‐effective operation [21, 22, 23, 24]. Moreover, electrochemical detection with digital signal readout effectively circumvents interference from the natural coloration of biological samples such as whole blood and urine. This inherent user‐friendliness renders it an ideal choice for the development of next‐generation smart home‐based testing platforms [25, 26].

Despite its promising attributes, the translation of electrochemical POCT technology into domestic settings has proceeded at a sluggish pace. A primary obstacle lies in the intricate matrix composition of unprocessed biological fluids, such as serum, saliva, and urine, which contain proteins, cellular debris, small‑molecule metabolites, and salts. These constituents tend to undergo nonspecific adsorption onto the working electrode surface, inducing irreversible interfacial fouling and thereby profoundly compromising assay specificity [27, 28, 29, 30]. A second concern is that signal stability and batch‑to‑batch reproducibility are difficult to ensure under non‑professional operation; frequent issues, including signal drift and elevated background noise, severely restrict the reliable quantification of low‑abundance biomarkers in a home environment. Furthermore, conventional electrochemical immunoassay strategies rely on immunological affinity recognition at the electrode interface coupled with electrical readout, which not only demands cumbersome conjugation of antibodies or aptamers but is also inherently constrained by the low efficiency of solid‑phase reactions. Therefore, although researchers have developed various anti‐fouling, portable electrochemical sensors, for example, Bagale et al. constructed a fouling‐resistant coating modified with zwitterionic copolymers and combined it with a graphene field‐effect transistor (gFET) biosensor to detect cTnI in untreated blood, yet it still struggles with cumbersome coating fabrication processes and complex steps involving coupling of interfacial bio‐capture elements, limiting its clinical universality and operational convenience [31, 32, 33, 34, 35]. Overall, devising novel electrochemical POCT strategies that resist complex matrix interference, deliver stable signals, and allow simple and efficient preparation and operation has become a critical bottleneck that must be overcome to advance these technologies toward practical home‑based applications.

**SCHEME 1:**
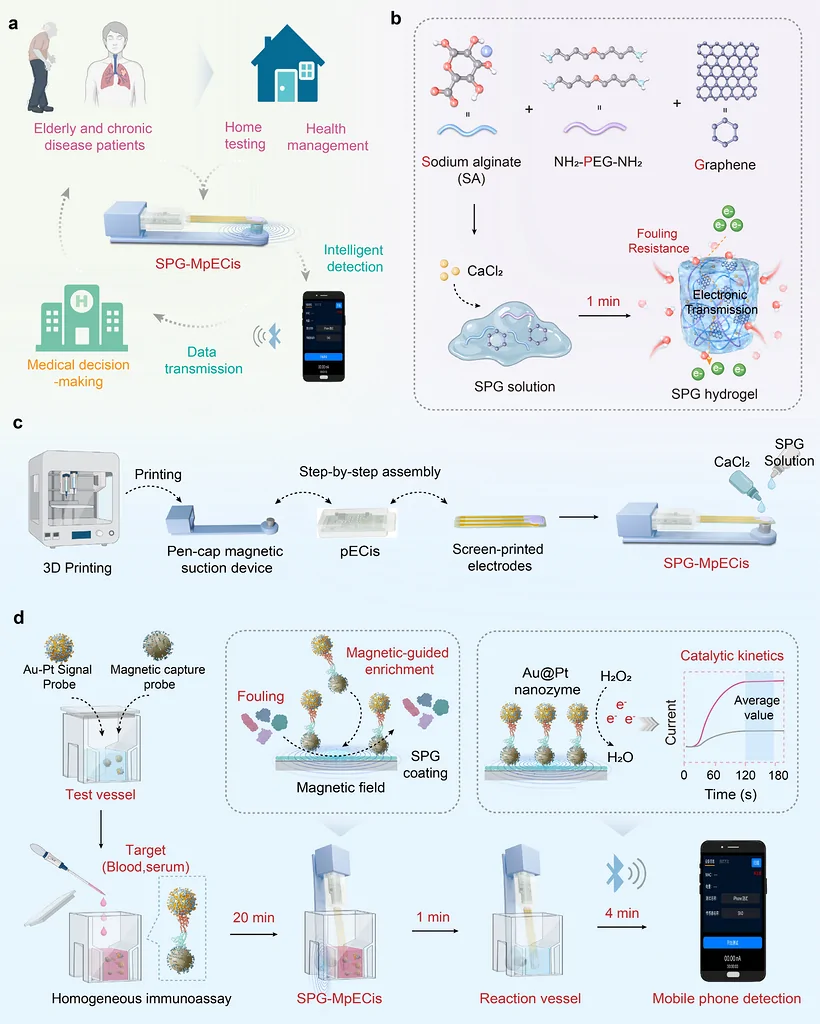
Schematic design and clinical application workflow of the integrated SPG‐MpECis platform. (a) Clinical home‐testing and wireless health management scenario. (b) Formulation and anti‐fouling mechanism of the conductive SPG hydrogel. (c) Structural assembly of the portable sensor using a custom 3D‐printed magnetic device. (d) Complete workflow from homogeneous target capture to smartphone‐based detection.

To address these challenges, we developed a magnetic portable electrochemical immunosensor (MpECis) based on a conductive and antifouling sodium alginate‐PEG‐graphene (SPG) hydrogel coating for quantitative biomarker analysis in undiluted serum (Scheme 1). Upon calcium‐ion triggering, the SPG solution undergoes alginate crosslinking within 1 min to form a stable hydrogel layer on the electrode surface [36]. The excellent hydrophilicity and steric repulsion of SPG hydrogel effectively suppress nonspecific adsorption from complex biological fluids such as serum and urine. Meanwhile, the embedded graphene ensures continuous electron‐transfer pathways throughout the hydrogel network, substantially reducing the interfacial resistance. Consequently, a plug‐and‐play MpECis device was assembled by integrating a 3D‑printed pen‐cap‐shaped magnetic guidance apparatus with a portable USB electrochemical workstation, eliminating the necessity for laborious electrode functionalization with antibodies. The homogeneous magnetic immunoassay format facilitates the directed enrichment of magnetic bead‐target‐metal nanozyme ternary complexes onto the SPG‐coated working electrode, where the metal nanozyme catalyzes substrate oxidation to generate a highly sensitive electrochemical readout. Notably, kinetic analysis of the metal nanozyme catalysis at the electrode interface revealed a signal plateau, prompting us to implement a time‐domain averaging strategy during this phase, which markedly improved signal reproducibility to a coefficient of variation (CoV) of 3.4%. This three‐pronged design, include rapid coating formation, magnetic enrichment, and signal‐stabilizing averaging, enabled the SPG‐MpECis platform to directly detect cardiac troponin I (cTnI) in serum from acute myocardial infarction (AMI) patients, extracellular vesicles (EVs) in the plasma of non‐small cell lung cancer (NSCLC) patients, and C‐reactive protein (CRP) in the fingertip blood of fever patients. The detection results show high concordance with clinical reference methods, and all measurements could be completed within 25 min. Overall, this work offers a novel electrochemical POCT strategy capable of direct analysis of complex clinical specimens and is expected to facilitate the translation of such technologies from laboratory prototypes to practical home‐based applications.

## 2 Results and Discussion

### 2.1 Rapid Fabrication and Performance Validation of the SPG Hydrogel Coating

**FIGURE 1:**
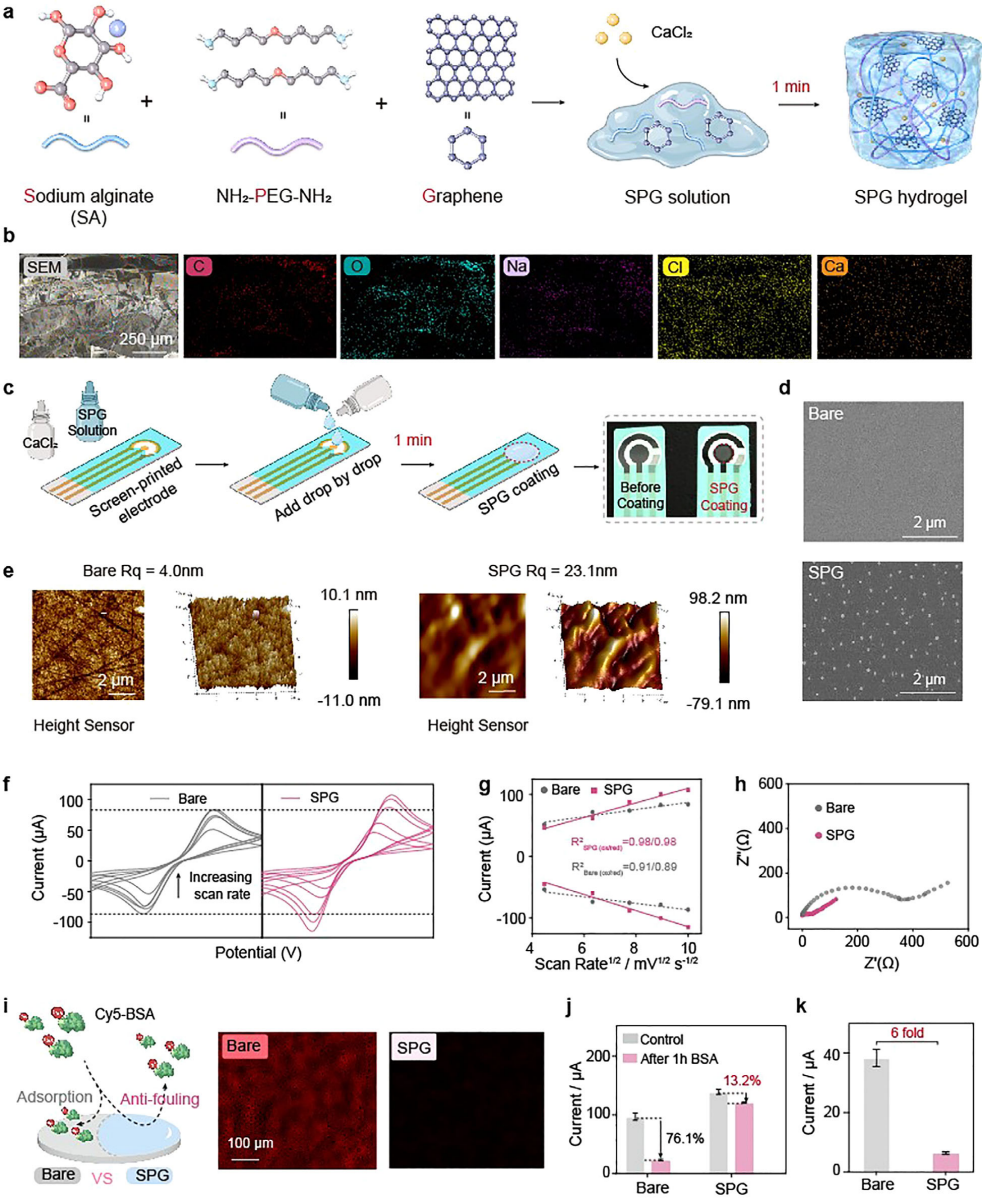
Preparation and characterization of SPG hydrogel coatings. (a) SPG precursor solution and hydrogel preparation process. (b) Scanning electron microscopy (SEM) images and energy‐dispersive spectroscopy (EDS) maps of the SPG hydrogel. (c) Method for preparing SPG hydrogel coatings on substrates. (d) Scanning electron microscope (SEM) images of the bare electrode and the SPG‐modified electrode. (e) Comparison of atomic force microscopy (AFM) topography and roughness between the bare substrate and the SPG hydrogel‐coated substrate. (f) Comparison of CV curves (left: bare electrode; right: SPG‐modified electrode). (g) The linear relationship between the current of the corresponding redox peak and the square root of the scan rate (v^1^/^2^). (h) Electrochemical impedance of the bare electrode and the SPG‐modified electrode. (i) Comparison of Cy5‐BSA fluorescence distribution between the bare electrode and the SPG‐modified electrode. (j) Comparison of DPV peak currents before and after a 1‐hour incubation of BSA on a bare electrode and SPG‐modified electrode. (k) Comparison of DPV peak currents after 1 hour's incubation of HRP on a bare electrode and SPG‐modified electrode. Data are presented as mean ± SD (n = 3). Statistical comparisons were performed using an unpaired Student's t‐test. A *p* value < 0.05 was considered statistically significant.

To avoid the complex covalent cross‐linking required in traditional electrochemical sensor coatings, this study has devised a conductive, anti‐fouling hydrogel coating (SPG) that is predicated on ion‐responsive rapid gelation. Its structural configuration is illustrated in Figure 1a. Sodium alginate (SA) functions as the gel network's structural foundation, polyethylene glycol (PEG) serves as the hydrophilic‐enhancing agent, and graphene (Figure S1) is integrated to establish internal conductive pathways. In essence, the rapid gelation process is facilitated by the ionic cross‐linking of SA, which is initiated by calcium ions. This mechanism enables the expeditious construction of an electrochemical sensing coating, integrating enhanced conductivity and hydrophilicity at a minimal cost. Initially, the rapid gelation capability of SPG in response to ions was verified. As demonstrated in Figure S3, the SPG solution undergoes a phase transition into a stable SPG hydrogel within 1 min of contact with calcium ions. Scanning electron microscopy (SEM) was utilized to preliminarily characterize the morphology and structure of the three hydrogel types (S, SP, SPG) constructed in a step‐by‐step manner (Figures 1b and S4). The results demonstrated that SPG manifests a uniform, porous, three‐dimensional network structure. Energy‐dispersive X‐ray spectroscopy (EDS) elemental mapping revealed that elements such as carbon (C), sodium (Na), chlorine (Cl), and calcium (Ca) are uniformly distributed within the gel (Figure S5). Furthermore, the FTIR results showed that compared to pure sodium alginate (S), SP and SPG exhibited a sharp C‐H stretching peak at 2880 cm^−^^1^ (originating from the ‐CH_2_CH_2_‐ backbone of PEG) and a significantly enhanced C─O─C ether bond stretching peak at 1100 cm^−^^1^, clearly confirming the successful incorporation of PEG. Moreover, the results of XPS full‐spectrum and relative elemental quantitative analysis show that, from the S hydrogel to the SPG hydrogel, the C/O ratio increases stepwise, while the Ca/C ratio decreases stepwise. This change is consistent with the experimental design of gradually introducing carbon‐rich components (PEG and graphene) during the preparation process, indicating that the SPG hydrogel integrates the characteristics of the three components at the elemental composition level. XRD analysis further revealed that the diffraction peaks (19°, 23°, 26°, 31°) attributed to the characteristic crystal structure in the SP hydrogel remained at the same positions after the introduction of graphene (SPG), but their intensities consistently decreased. These results indicate that the introduction of graphene did not alter the original crystal structure type, and that the SPG retains the crystalline phase characteristics of the SP hydrogel (Figure S6). Subsequently, through precise volume control, an ultrathin SPG hydrogel coating was successfully prepared at the screen‐printed electrode interface (Figure 1c), thereby reducing the resistance signal caused by coating thickness. Concurrently, SEM images substantiated the successful deposition of the SPG hydrogel on the electrode surface. In comparison to the bare electrode, a uniform embedding of flake‐like nanomaterials was observed on the SPG‐modified electrode surface (Figures 1d and S7). AFM characterization results further confirmed the formation of the coating. As demonstrated in Figure 1e, the surface roughness of the SPG‐coated surface exhibited a substantial increase (Rq = 23.1 nm) in comparison to the bare glass slide substrate (Rq = 4 nm). A systematic comparison of the SPG coating fabrication strategy with various sensor interface preparation methods reported in the literature revealed that, in contrast to other methods that typically require multi‐step, time‐consuming processes, the method proposed in this study requires only a single step to complete the in situ construction of the sensor coating within 1 min, thereby significantly simplifying the electrode modification process (Table S1).

In addition, a systematic evaluation was conducted to assess the electrochemical signal output performance and signal stability in complex matrices. Potassium ferricyanide was utilized as the working solution in cyclic voltammetry (CV) tests conducted on bare electrodes and SPG‐modified electrodes at varying scan rates. As demonstrated in Figure 1f, when the scan rate exceeded 40 mV/s, the SPG‐modified electrode exhibited significantly higher redox peak currents at the same scan rate. Further analysis indicates that the peak current of the SPG‐modified electrode exhibits a good linear relationship with the square root of the scan rate (Figure 1g), suggesting that the redox process at the SPG‐modified electrode is closer to ideal reversible diffusion control (*SPG: R^2^ = 0.98/0.98; Bare: R^2^ = 0.91/0.89*). Moreover, electrochemical impedance spectroscopy (EIS) analysis (Figure 1h) demonstrated that the charge transfer resistance (*Rct*) of the SPG‐coated electrode was considerably lower than that of the bare electrode. The findings suggest that, likely due to graphene's excellent conductivity, the SPG coating effectively reduces charge transfer resistance at the electrode interface, significantly enhancing the electrode's conductivity. This enhanced electrochemical performance is crucial for ensuring high sensitivity in subsequent detection. In addition to good electrical conductivity, maintaining stable electrochemical signal output in complex matrices is equally important. In order to verify the anti‐fouling performance of the SPG coating, bovine serum albumin (BSA) and natural horseradish peroxidase (HRP) were utilized as model contaminants. A systematic evaluation was conducted to assess the signal stability of the SPG‐modified electrode within a single‐protein environment, a process that involved the integration of fluorescence imaging with electrochemical methods. As demonstrated in Figure 1i, in comparison with the bare substrate, the fluorescence signal of Cy5‐labeled BSA on the SPG‐coated surface was notably diminished. Moreover, differential pulse voltammetry (DPV) testing further revealed that, compared to their respective controls, the current signal of the bare electrode declined by 76.1% following 1 h of incubation in a BSA solution, whereas that of the SPG‐modified electrode decreased by a mere 13.2% (Figure 1j).

When HRP was used as the model contaminant (Figure 1k), the DPV current response of the bare electrode in the hydrogen peroxide working solution was approximately six times that of the SPG‐modified electrode, indicating a significant difference between the two. The collective outcomes of these experiments suggest that the SPG coating effectively suppresses the nonspecific adsorption of proteins on the electrode surface, thereby ensuring the electrochemical signal stability of the sensor within a single‐protein environment.

In summary, the rapid ion‐responsive cross‐linking properties of sodium alginate enable the expeditious fabrication of SPG coatings in non‐traditional settings, thereby ensuring excellent on‐site deployability and application flexibility. Concurrently, this sensing interface exhibits a notable degree of resistance to nonspecific adsorption while concurrently demonstrating stable electrochemical response characteristics. This combination of properties positions it as a promising candidate for achieving high sensitivity and reliable signal output in the detection of biological samples.

### 2.2 Investigation of the Conductivity and Anti‐Fouling Mechanisms of SPG Hydrogel Coatings

Previous studies have demonstrated that SPG hydrogel coatings exhibit significant advantages in terms of formation rate, electrical conductivity, and anti‐fouling performance. However, to achieve optimal detection performance, further optimization of the ratio of coating components is necessary. In addition, the mechanism of synergy among the components in this SPG coating system—specifically, the configuration of the conductive pathways—as well as the physicochemical basis for enhanced anti‐fouling capabilities, has yet to be systematically elucidated. To this end, this chapter systematically optimizes the component ratios of the SPG coating and thoroughly investigates its conductive and anti‐fouling mechanisms, aiming to lay a theoretical foundation for subsequent highly sensitive detection.

**FIGURE 2:**
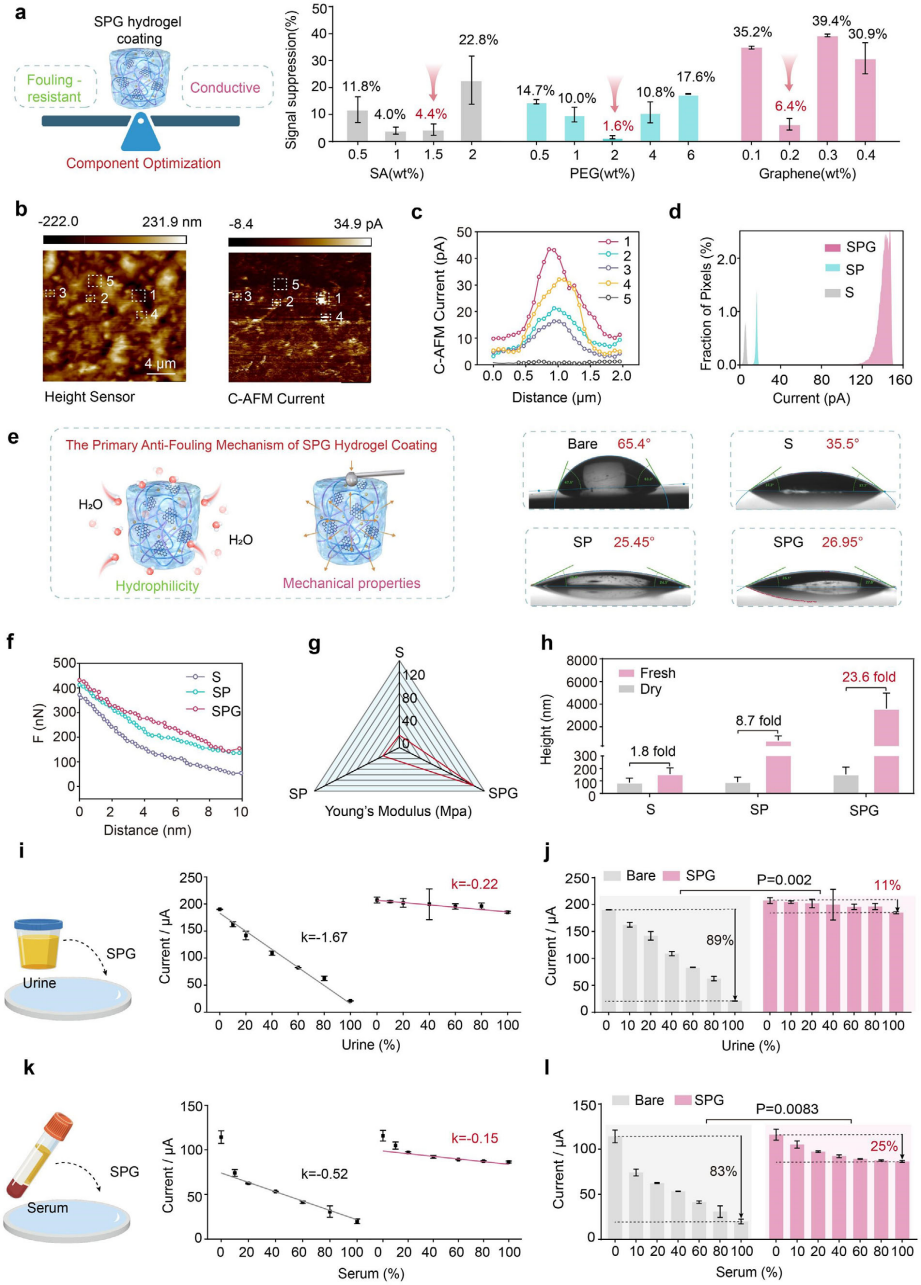
Investigation of the conductive and anti‐fouling mechanisms of the SPG‐modified electrode. (a) Component optimization of SPG‐modified electrode. (b) AFM topography (left) and C‐AFM current map (right); (c) Current response curves from points 1 to 5 in the C‐AFM image. (d) Overall current response distribution map of S, SP, and SPG coatings based on C‐AFM images. (e) Schematic diagram of the SPG stain‐resistant mechanism and water contact angle tests on bare electrodes and S, SP, and SPG coatings. (f,g) Force‐displacement curves obtained by AFM probe measurement of S, SP, and SPG coatings, and quantitatively calculated Young's modulus results. (h) Comparison of the thicknesses of S, SP, and SPG coatings in fresh and dried states. Evaluation of the antifouling performance of SPG‐modified electrode in complex biological matrices. (i–k) Linear fitting of DPV response currents against m = atrix concentration. (j–l) Comparison of DPV response currents for bare electrode and SPG‐modified electrode. after 1‐hour incubation in urine and serum at different concentrations. Data are presented as mean ± SD (n = 3).

First, the ratio of the three components in the SPG was systematically optimized. Given that albumin is the most abundant non‐conductive protein in serum, its nonspecific adsorption on the electrode surface can severely hinder interfacial electron transfer, leading to attenuation of the current signal. Consequently, the current signal suppression rate was utilized as an evaluation metric before and after incubation with BSA to identify the optimal component ratio. As demonstrated in Figure 2a, when the concentrations of sodium alginate (SA), polyethylene glycol (PEG), and graphene were 1 wt%, 2 wt%, and 0.2 wt%, respectively, the SPG‐modified electrode exhibited the lowest signal suppression rate, indicating optimal resistance to protein adsorption. However, when the SA concentration was 1 wt%, the low cross‐linking density of the gel network formed by the low‐concentration SA likely weakened the adhesion between the coating and the substrate, causing the coating to easily peel off the electrode surface during incubation. Consequently, 1.5 wt% was selected as the SA concentration for subsequent experiments to balance the structural stability and anti‐fouling performance of the coating.

Consequently, we employed conductive atomic force microscopy (C‐AFM) to conduct a preliminary investigation into the conductive mechanism of the SPG coating. As illustrated in Figure 2b, the AFM topography of the SPG coating surface is presented alongside the corresponding C‐AFM current image. A thorough analysis of the C‐AFM images revealed the presence of discretely distributed high‐current‐response regions on the coating surface. The absence of discernible corresponding structures in the topography image suggests that these regions originated from the localized conductive properties of the graphene components within the SPG hydrogel. Conversely, the C‐AFM images of the pure S and SP hydrogel coatings, devoid of graphene, exhibited uniform and low current signals, thereby demonstrating typical insulating behavior (Figure S9). Subsequent quantitative analysis (Figure 2c) demonstrated that the high‐current‐response points (points 1–4) within the SPG coating exhibited considerably elevated current values in comparison to the non‐conductive regions (point 5). Furthermore, a comparative analysis of the cross‐sectional current distributions of the three hydrogel coatings (Figure 2d) revealed that the SPG coating demonstrated a substantially higher overall current level compared to the S and SP groups. The outcomes of this study demonstrate that the uniform dispersion of graphene within the SPG hydrogel matrix confers two key benefits. First, it provides excellent electrical conductivity to the coating. Second, it establishes efficient localized conductive pathways within the insulating SA/PEG matrix. These pathways contribute to the formation of a macroscopic conductive network that extends throughout the entire coating.

In order to investigate the anti‐adsorption mechanism of the SPG coating, an AFM probe was utilized as a simulated contaminant, and the coating's ability to repel foreign particles was evaluated by recording force‐displacement curves (Figure S10). As demonstrated in Figure 2f, as the AFM probe gradually approached the coating surface from a distance of 10 nm, the overall repulsive force experienced by the probe in the SPG group was significantly higher than that in the S and SP groups. This finding indicates that SPG exhibits stronger repulsion against foreign particles. Furthermore, as demonstrated in Figure 2g, the calculated Young's modulus from these data was 120 MPa for the SPG group, which is significantly higher than that of the S and SP hydrogel coatings, aligning with prior reports on the mechanical performance of graphene‐reinforced hydrogels [37, 38]. Consequently, it can be deduced that the enhanced mechanical properties of SPG are attributable to the incorporation of graphene. Subsequently, a comprehensive evaluation of the hydrophilic properties of each hydrogel coating was conducted. This evaluation involved the combination of water contact angle measurements (Figure 2e) with changes in coating thickness under dry and swollen conditions (Figure S11). As demonstrated in Figure 2h, SPG demonstrated the most significant swelling capacity, with its thickness increasing 23.6‐fold compared to its dry state. The introduction of the hydrophilic molecule PEG, as anticipated, led to an enhancement in the overall hydrophilicity of the coating. Specifically, the swelling thickness of SP increased to 8.7‐fold, which is notably higher than the 1.8‐fold increase observed in S. However, the water absorption performance of SP remained significantly lower than that of the SPG group. According to Zhao and co‐workers [36], the incorporation of graphene can induce anisotropic swelling in the gel coating, thereby significantly enhancing its volumetric expansion capacity. Conversely, in the absence of graphene, the gel coating exhibits nearly isotropic swelling behavior. Consequently, the SPG coating, with its enhanced mechanical properties and superior swelling capacity, can form a thicker hydrophilic layer and generate a stronger steric hindrance effect, thereby more effectively resisting the adhesion of external contaminants. Finally, to further evaluate the applicability of the SPG coating in real biological matrices, we investigated its electrochemical signal behavior in complex body fluids such as serum and urine. The results of the study indicate that the SPG‐modified electrode exhibits superior signal stability compared to the bare electrode in both media. An analysis of the linear relationship between signal intensity and body fluid concentration (Figure 2i,k) reveals that the current decay of the SPG‐modified electrode is relatively gradual, with fitted slopes (k) of ‐0.22 and ‐0.15 in urine and serum, respectively. In contrast, the current decline of the bare electrode was more pronounced, with corresponding slopes of ‐1.67 and ‐0.52, indicating that its signal is more susceptible to interference from complex matrices. A subsequent comparison of peak current values at varying concentrations (Figure 2j,l) indicated that, in undiluted urine and serum, the peak current of the bare electrode diminished by 89% and 83%, respectively, while that of the SPG‐modified electrode diminished by only 11% and 25%, respectively. The collective outcomes of these experiments demonstrate the efficacy of the SPG coating in suppressing nonspecific adsorption of the electrode in real biological fluids. The results also indicate a delay in signal decay and the endowment of the SPG‐modified electrode with effectively resistance to interference and signal stability.

In summary, the SPG‐modified electrodes exhibit several noteworthy advantages. First, they exhibit a highly efficient conductive network formed by graphene in the SPG hydrogel coating. Second, they demonstrate a synergistic enhancement of hydrophilicity and spatial repulsion effects resulting from anisotropic swelling and improved mechanical properties. Third, they effectively resist the nonspecific adsorption of individual proteins. Finally, they demonstrate electrochemical signal stability in complex biological environments such as serum and urine. This provides a solid foundation for achieving highly sensitive and accurate electrochemical detection in complex real‐world samples, demonstrating strong potential for point‐of‐care applications.

### 2.3 Development of an MpECis System Based on SPG Hydrogel Coatings

Conventional electrochemical detection workflows suffer from two major drawbacks. First, electrode interface functionalization relies on a time‑consuming, multistep antibody immobilization procedure. Second, target capture requires protracted incubation and multiple washing steps on solid‐phase surfaces, rendering the process cumbersome and error‑prone. Taking gold electrodes as a representative example, interface functionalization typically involves self‐assembly of thiol molecules to form a self‐assembled monolayer (SAM), followed by covalent antibody attachment and blocking of residual active sites, a sequence that often exceeds 5 h in total. Meanwhile, the target capture stage demands sequential antigen‐antibody binding on the solid phase, accompanied by repeated washing steps, requiring at least 40 min. By contrast, magnetically guided technology shifts these multistep, time‑intensive operations into a simple and efficient homogeneous solution, where magnetic field‑driven enrichment rapidly concentrates immune complexes onto the electrode surface, compressing the overall assay time from hours to minutes. Building on these insights, we developed the SPG‐MpECis platform, which seamlessly integrates “homogeneous incubation, magnetically controlled enrichment, and electrochemical readout” into a unified system, yielding a detection strategy particularly suited for rapid on‑site deployment.

**FIGURE 3:**
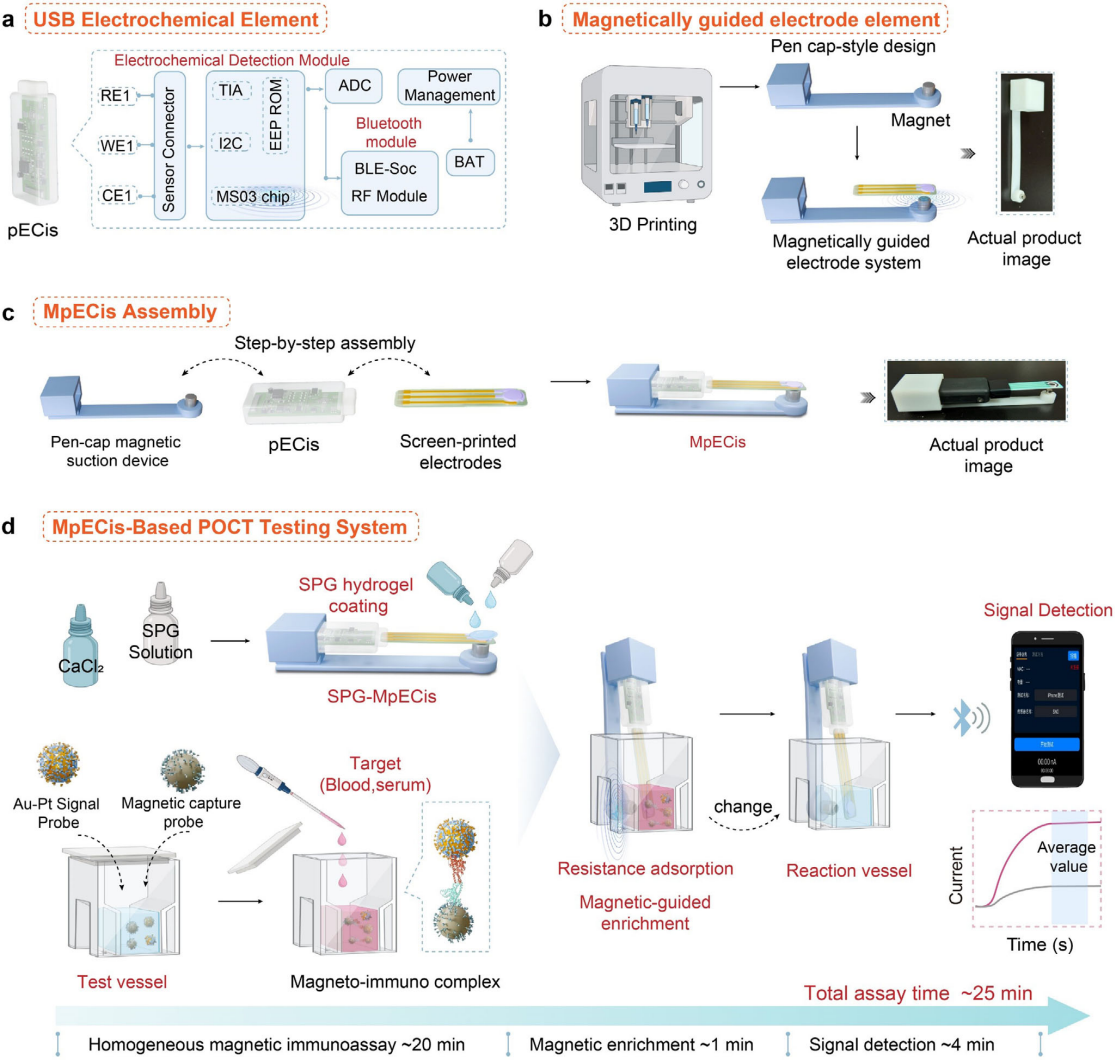
Design and workflow of the integrated SPG‐MpECis system for POCT applications. (a) Schematic illustration of the portable electrochemical detection module (USB Electrochemical Element). (b) Schematic illustration and photograph of the custom‐designed 3D‐printed pen‐cap magnetic suction device. (c) MpECis assembly process and actual product image. (d) Schematic of the SPG‐MpECis‐based POCT testing system.

Firstly, we have integrated a portable electrochemical detection module (USB Electrochemical Element) based on a design by our partner team, Shenzhen Refresh Biosensing Technology Co., Ltd. This module integrates a sensor interface, a TIA amplifier, an ADC converter, and a Bluetooth transmission unit, facilitating wireless signal reading and cloud data synchronization via a smartphone (Figure 3a). A comparison of different electrode platforms reveals significant discrepancies in their magnetic control capabilities. Traditional magnetic electrochemical immunosensor (MECis) offers a convenient solution, as they allow magnets to be securely fixed within the glassy carbon electrode. In contrast, the screen‐printed electrode (SPE) utilized in the portable electrochemical immunosensor (pECis) lacks a built‐in magnetic structure, necessitating the incorporation of an external magnet that can be challenging to secure. To address this, a 3D‐printed pen‐cap‐style magnetic mold was designed and customized (Figures 3b, S16), enabling the SPE in the portable pECis to achieve magnetic enrichment capabilities comparable to those of traditional magnetic glassy carbon electrodes. The innovation of this mold is characterized by the following aspects: The pen‐cap‐style structural design forms a precise “pen‐cap‐to‐pen‐tip” fit with the main USB drive unit of the SPG‐pECis, ensuring secure fixation. The cylindrical magnet embedded at the rear end of the mold is precisely aligned with the working electrode region of the SPE, enabling rapid enrichment of the immunomagnetic beads dispensed onto the electrode surface. The integration of this mold with the SPG‐pECis resulted in the successful construction of an integrated SPG‐MpECis (Figure 3c), thereby effectively addressing the functional gap in magnetic‐controlled detection for portable pECis.

The development of a detection system was informed by the aforementioned magnetically controlled electrode platform, and it employs a “homogeneous incubation‐magnetically controlled enrichment‐electrochemical readout” approach (Figure 3d). The system is composed of three primary modules. The reaction module contains lyophilized powders of immunomagnetic beads and immuno AuPt nanozymes. It is used for homogeneous target recognition and signal probe labeling. The successful formation of the magnetic bead‐target‐AuPt immunocomplex was validated by first preparing immunomagnetic beads (Figure S13) and AuPt nanozymes with uniform morphology and elemental composition (Figure S14). Effective antibody conjugation was confirmed through the enhancement of the 280 nm UV absorption peak. Subsequent TMB colorimetric assays demonstrated that the immune complex successfully assembled and catalyzed color development exclusively in the presence of the target, generating a distinct characteristic absorption peak at 650 nm (Figure S15a–c). These results suggest that the magnetically controlled immune complex system was successfully established, thereby laying the foundation for subsequent highly sensitive detection based on SPG‐MpECis. (2) Electrode preparation module: The organization provides screen‐printed electrodes, SPG solution, calcium chloride solution, and MpECis components for the rapid on‐site assembly of SPG‐MpECis. (3) Detection module: The kit includes a PBS buffer and an H_2_O_2_ working solution for signal output.

The operational workflow of the SPG‑MpECis system proceeds as follows. First, as little as 10 µL of finger‑prick blood or plasma is added to a reaction cup containing magnetic capture probes and metal nanozyme signaling probes, where the ternary immune complexes form within 20 min. Concurrently, the SPG hydrogel is rapidly coated onto the screen‑printed electrode of the MpECis in just 1 min. The electrode is then inserted into the same reaction cup, and a magnetic field guides the directed enrichment of the immune complexes onto the electrode surface. Finally, the SPG‐MpECis is transferred to a detection cup containing H_2_O_2_, where the AuPt nanozymes catalyze the oxidation of H_2_O_2_ to generate an electrochemical signal. Through this streamlined procedure, non‑professionals can complete the entire home‑based test within 25 min, and the resulting signal can be conveniently read out and interpreted via a smartphone.

### 2.4 Investigation of the SPG‐MpECis Signal Output Mode

Differential pulse voltammetry (DPV) is a method of signal generation that involves the application of a pulse sequence and the implementation of differential sampling. The response values obtained through this process exhibit a high degree of dependence on the temporal alignment between the applied potential and the enzymatic reaction process. Given the dynamic nature of the rate of enzymatic reactions over time, the instantaneous current recorded by DPV at the pulse sampling moment fluctuates, resulting in inconsistent response signals for the same sample at different detection time points. Additionally, DPV is generally employed for endpoint detection, which hinders the attainment of continuous, real‐time signal monitoring and precludes the comprehensive capture of the dynamic evolution of reaction kinetics. This signal instability poses a significant challenge to the accurate quantification of clinical samples. Therefore, establishing a standardized testing protocol and identifying the optimal detection time window are key prerequisites for ensuring the reliability, reproducibility, and clinical applicability of test results.

**FIGURE 4:**
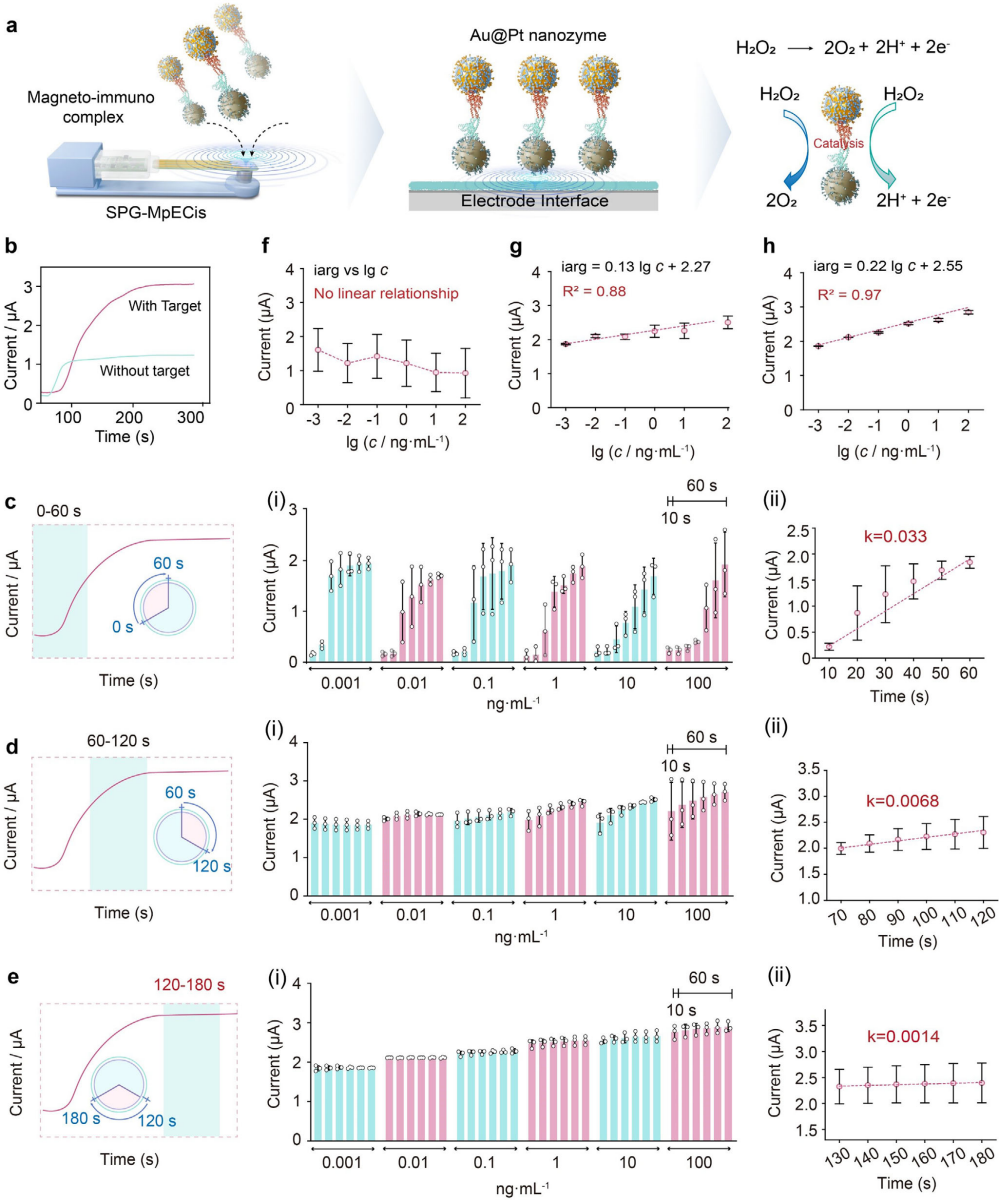
Detection performance evaluation of cTnI based on SPG‐MpECis integrated portable detection platform sensor. (a) Schematic diagram of the sandwich immunoassay system and the enzymatic reaction. (b) Feasibility of detecting cTnI based on SPG‐MpECis. Validation of the SPG‐MpECis detection performance: Analyze the (c‐h) linearity of the cTnI electrical signals measured at 10 s intervals within the three‐time windows of 0–60 s, 60–120 s, and 120–180 s. Data are presented as mean ± SD (n = 3).

Initially, cTnI was employed as a model target to assess the viability of the SPG‐MpECis detection strategy (Figure 4b). The detection mechanism is illustrated in Figure 4a. First, the magnetic bead‐target‐AuPt immunocomplex is magnetically enriched onto the electrode surface. Then, the AuPt nanozyme catalyzes a two‐electron oxidation reaction of H_2_O_2_ (H_2_O_2_ → 2O_2_+ 2H^+^ + 2e^−^) under an applied oxidizing potential. This generates a cathodic reduction current. Given that each target molecule is labeled with multiple AuPt nanozymes, this catalytic reaction achieves significant signal amplification. That is to say, the current intensity is positively correlated with the target concentration. Subsequent to this, the conditions for immunocomplex formation were systematically optimized (Figure S17). Subsequently, we expanded our investigation to encompass the signal output characteristics of SPG‐MpECis. The experimental findings demonstrated that the detection signal of SPG‐MpECis manifests distinct temporal dependencies. Consequently, the measurement of the same sample at varying temporal points may result in dissimilar current response values. This phenomenon can be attributed to the kinetic characteristics of the enzymatic reaction mediated by the AuPt nanozyme. The catalytic reaction rate of the nanozyme undergoes dynamic changes over time, leading to a nonlinear evolution of the signal output. Consequently, to establish a standardized and reliable detection protocol, it is necessary to identify the optimal detection time window. In light of these findings, the 0–180 s detection cycle was methodically divided into three consecutive time intervals for systematic comparison. The initial interval ranged from 0 to 60 s, followed by a subsequent range of 60 to 120 s, and finally, an additional range of 120 to 180 s. As illustrated in Figure S18, the current signals were obtained by detecting targets of varying concentrations at 10 s intervals within each phase. Figure 4c–e further illustrated the dynamic trends of current signals over time for each phase. It is noteworthy that the gradient of the fitted straight line experiences a gradual decline as the detection time progresses, indicating that signal fluctuations are minimal during the 120–180 s phase. This suggests that the measured values are closest to the true levels during this period. Subsequent analysis of the correlation between target concentration and current response across these phases revealed that during the 0–60 s phase, the two exhibited a nonlinear relationship; whereas during the 60–120 s and 120–180 s phases, both phases demonstrated a good linear relationship. Among these, the linear fit in the 120–180 s window demonstrated the highest goodness of fit (*R^2^ = 0.97*), indicating that the current signal's response to target concentration exhibited the strongest linear correlation within this time window, making it most suitable for quantitative analysis (Figure 4f–h). Additionally, a comprehensive evaluation was conducted to assess the detection specificity, stability, and reproducibility across the three designated time windows (Figure S19). The findings indicated that the 120–180 s interval demonstrated optimal performance in all metrics. In summary, the optimal detection time window was determined to be 120–180 s. Within this time frame, the current signal demonstrates optimal stability and concentration interpretability, providing a clear basis for standardizing subsequent detection procedures.

### 2.5 SPG‐MpECis for Early Home Monitoring of AMI

Based on the established standardized testing protocol, this study further evaluates the clinical utility of the developed SPG‐MpECis for the rapid auxiliary diagnosis of acute myocardial infarction (AMI). cTnI, a highly sensitive and specific biochemical marker of myocardial injury, is widely used in clinical practice, as elevated serum levels serve as a key indicator of myocardial damage [39]. Since circulating cTnI in blood exists mainly as a cTnI‐cTnC complex, this assay system utilizes antibodies targeting its stable central domain to reliably capture both free and complexed cTnI. The implementation of rapid and accurate cTnI testing has been demonstrated to result in a substantial reduction in the diagnostic time for patients experiencing acute chest pain. This, in turn, ensures that the critical window for early reperfusion therapy is maintained [40].

**FIGURE 5:**
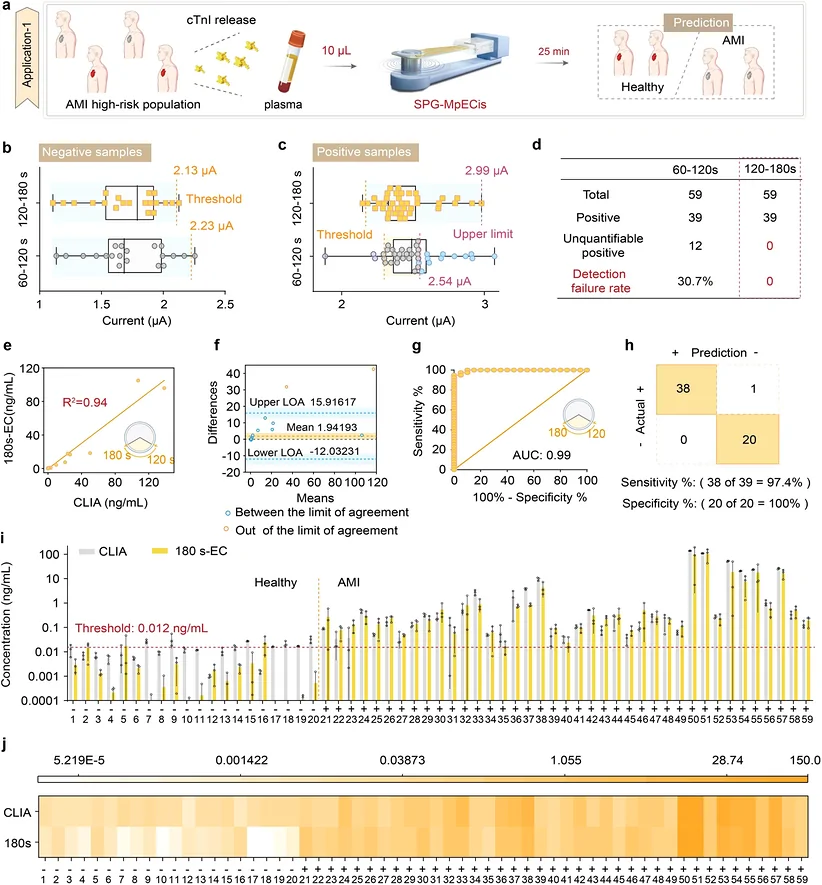
(a) Performance validation of the SPG‐MpECis for the detection of cTnI in clinical serum samples. (b) Detection signal distribution of negative clinical samples in 60–120 s and 120–180 s time windows. (c) The detection signal distribution of positive clinical samples in 60–120 s and 120–180 s time windows. (d) Detection failure rate of cTnI clinical samples detected by SPG‐MpECis at different time windows. (e) Correlation and (f) consistency analysis between the detection results of SPG‐MpECis within 120–180 s and the clinical samples of cTnI detected by the CLIA method. (g‐h) The diagnostic performance of SPG‐MpECis for cTnI within 120–180 s was evaluated according to the ROC curve and confusion matrix at time intervals. Comparative analysis of SPG‐MpECis detection results within 120–180 s and CLIA results: (i‐j) bar graph and heat map. Data are presented as mean ± SD (n = 3).

The SPG‐MpECis system was utilized to conduct cTnI testing on a total of 59 clinical serum samples, encompassing 20 healthy controls and 39 AMI patients (Figure 5a for a visual representation of this data). The study collected test results from two time windows (60‐120 s and 120–180 s) and compared them with those from the clinical gold standard method, chemiluminescent immunoassay (CLIA). Initially, an analysis was conducted to ascertain the distribution of detection signals for clinical samples within the two designated time windows. As demonstrated in Figure 5b, in both the 60–120 s and 120–180 s time windows, the detection signals for negative samples remained consistently below the diagnostic threshold, and there was no significant difference between the two windows. As demonstrated in Figure 5c, within the 60–120 s interval, the detection signals of a substantial number of positive samples surpassed the upper limit of the linear detection range (blue dots), while the signals of certain positive samples remained below the detection threshold (purple dots). Conversely, within the 120–180 s interval, the signals of all positive samples were concentrated within the linear range, signifying that the response within this window was more stable and reliable. A subsequent analysis of the detection failure rate (Figure 5d) indicated that among the 39 positive samples collected in the 60–120 s window, 12 samples exceeded the upper limit of the linear range (detection failure rate of 30.7%), whereas all positive samples collected in the 120–180 s window fell within the linear range (detection failure rate of 0%). The findings suggest that the 60–120 s time frame is susceptible to severe signal saturation issues and does not meet the reliability requirements for clinical testing. Conversely, the signal response exhibited stability within the 120–180 s time window, with all positive samples falling within the linear detection range, thereby satisfying the fundamental criteria for clinical quantitative analysis. Consequently, a detection time window ranging from 120 to 180 s was identified as the optimal range for SPG‐MpECis. A systematic comparison was then conducted between the results obtained from this method and those derived from the clinical standard method, CLIA, within this specified time window.

We then proceeded to evaluate the accuracy and consistency of SPG‐MpECis within the 120–180 s window. As demonstrated in Figure 5e, the SPG‐MpECis outcomes exhibited a robust positive correlation with the CLIA method (*R^2^ = 0.94*). Bland‐Altman analysis further demonstrated good agreement between the two methods (Figure 5f). Finally, the diagnostic performance of SPG‐MpECis within the 120–180 s window was evaluated. Receiver operating characteristic (ROC) curve analysis (Figure 5g) demonstrated an area under the curve (AUC) of 0.99; confusion matrix analysis (Figure 5h) indicated corresponding sensitivity and specificity of 97.4% and 100%, respectively. The bar charts and heatmaps in Figure 5i,j provide a more comprehensive summary of the comparison of detection results between the SPG‐MpECis and the CLIA method for all 59 samples (with a diagnostic threshold set at 0.012 ng/mL). These visual representations offer a quantitative illustration of the agreement between the two methods.

### 2.6 SPG‐MpECis for Early Home Monitoring of NSCLC

In order to expand the range of potential applications of SPG‐MpECis, its performance in the context of liquid biopsy detection was evaluated. Liquid biopsy, a non‐invasive cancer screening strategy, has garnered considerable attention in recent years. However, circulating tumor cells (CTCs) are scarce in the early stages of cancer, and their efficient capture and enrichment remain challenging [41]. Circulating tumor DNA (ctDNA) is released in limited quantities when tumor burden is low. The short half‐life of ctDNA and its susceptibility to dilution by background DNA can lead to false‐negative results [42]. In contrast, EVs are present in the blood and are continuously released even in the early stages of cancer, thereby demonstrating unique potential advantages in early cancer diagnosis.

**FIGURE 6:**
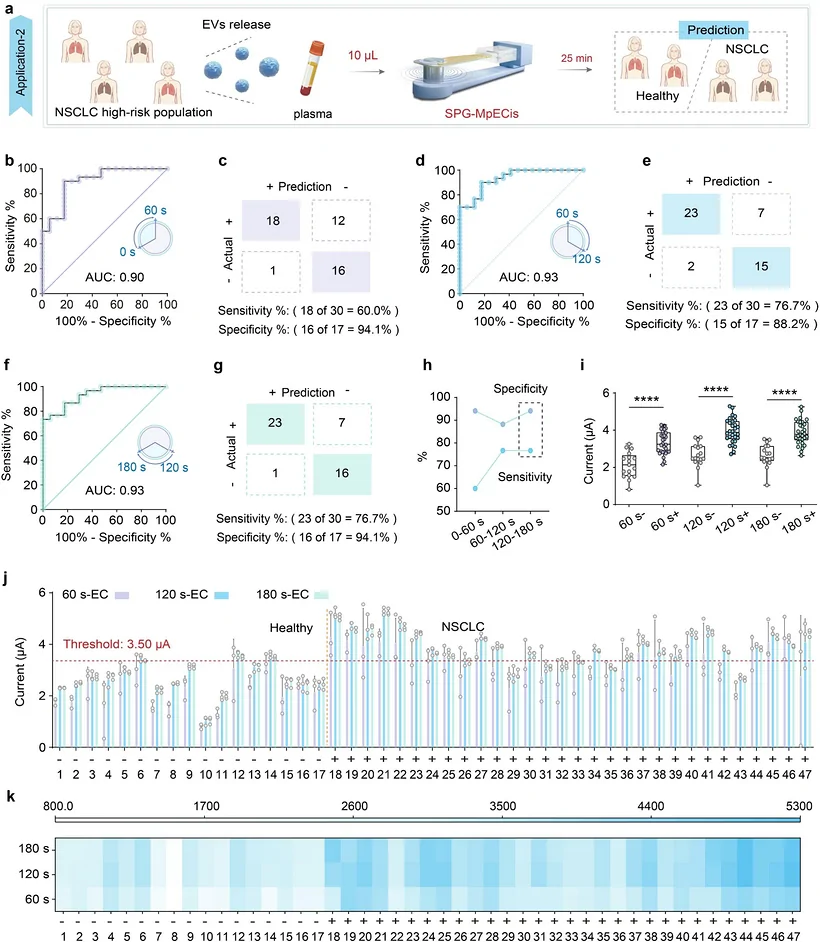
(a) Performance validation of the SPG‐MpECis for the detection of EVs in clinical serum samples. Evaluation of the SPG‐MpECis's diagnostic performance by time interval based on ROC curves and confusion matrices: (b,c) 0–60 s; (d,e) 60–120 s; (f,g) 120–180 s. (h) Sensitivity and specificity of the SPG‐MpECis in detecting EVs at different time points. (i) Box plots of the SPG‐MpECis's EVs detection results at different time intervals. (j) Bar charts and (k) heatmaps showing the SPG‐MpECis's EV detection results across three time periods. Data are presented as mean ± SD (n = 3). Statistical significance was indicated as *p < 0.05, **p < 0.01, **p < 0.001.

Consequently, a rapid detection method for plasma EVs in patients with non‐small cell lung cancer (NSCLC) was established (Figure 6a), and a systematic evaluation of its fundamental detection performance was conducted. In accordance with the previously delineated optimization strategy, the 0–180 s detection window was meticulously subdivided into three consecutive time intervals for systematic comparison. The initial interval ranged from 0 to 60 s, followed by a subsequent range of 60 to 120 s, and finally, an additional range of 120 to 180 s. As illustrated in Figures S20a–c and S21, the current signals obtained from detecting EVs at varying concentrations every 10 s within each phase are presented. The dynamic trends of current signals over time exhibited favorable linear relationships in all phases, and the fitted straight lines for the 60–120 s and 120–180 s phases had lower slopes, indicating minimal signal fluctuations and measurements close to the true values. Subsequent analysis of the correlation between target concentration and current response in each phase revealed that the 0–60 s phase exhibited a nonlinear correlation, whereas both the 60–120 s and 120–180 s phases demonstrated a strong linear relationship (*R^2^ = 0.99*). In addition, a comprehensive evaluation was conducted to assess the detection specificity, stability, and reproducibility across the three designated time windows (Figure S20d–g). The findings indicated that the 120–180 s phase exhibited the optimal overall performance across these metrics. Accordingly, we undertook the detection and analysis of serum EV samples, employing a total of 47 clinical specimens (comprising 17 healthy controls and 30 NSCLC patients). As demonstrated in Figure 6b–g, a comprehensive set of detection results was collected from the three designated time windows (0–60 s, 60–120 s, and 120–180 s). These results underwent rigorous analysis, including the construction of ROC curves and the generation of confusion matrices. The results demonstrated that the area under the curve (AUC) for the 0–60 s window was 0.90, with sensitivity and specificity of 60.0% and 94.1%, respectively. For the 60–120 s window, the AUC increased to 0.93, with a sensitivity of 76.7% and specificity of 88.2%. For the 120–180 s window, the AUC was 0.93, with sensitivity and specificity of 76.7% and 94.1%, respectively. Figure 6h further compares the sensitivity and specificity across the three‐time windows, confirming that the 120–180 s window exhibits optimal diagnostic performance. Figure 6i–k provided a comprehensive summary of the measurement results for the 47 EV samples across the three aforementioned time windows.

### 2.7 SPG‐MpECis for Early Home Monitoring of Inflammatory Disorders

**FIGURE 7:**
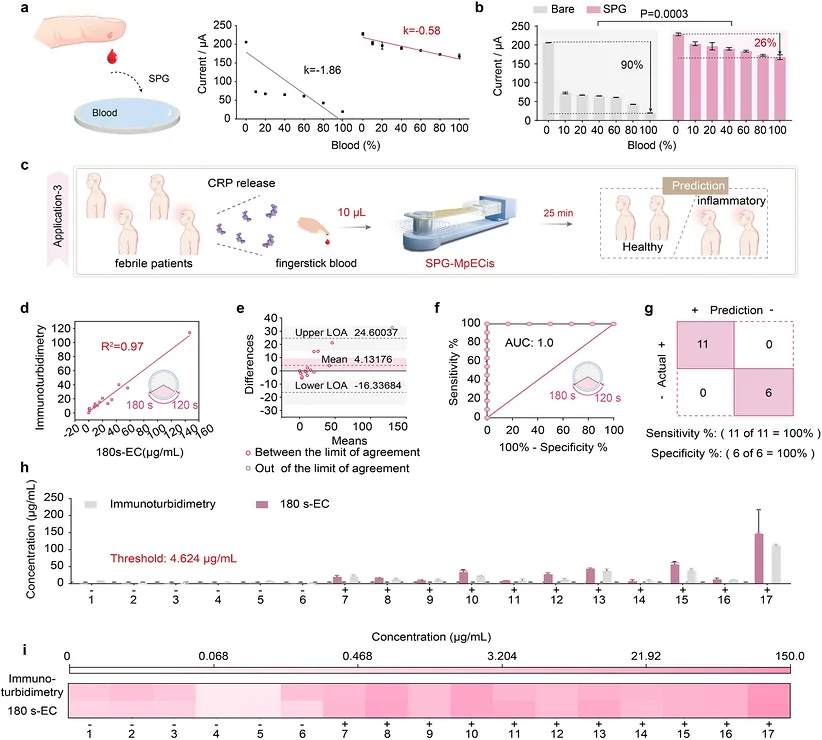
(a) Linear fitting of DPV response currents against matrix concentration. (b) Comparison of DPV response currents for bare electrode and SPG modified electrode after 1 hour incubation in finger‐prick blood at different concentrations. (c) Performance validation of the SPG‐MpECis for the detection of CRP in finger‐prick blood samples. (d) Correlation and (e) consistency analysis between the detection results of SPG‐MpECis within 120–180 s and the clinical samples of CRP detected by the immunoturbidimetry method. (f,g) The diagnostic performance of SPG‐MpECis for CRP within 120–180 s was evaluated according to the ROC curve and confusion matrix at time intervals. Comparative analysis of SPG‐MpECis detection results within 120–180 s and immunoturbidimetry results: (h,i) bar graph and heat map. Data are presented as mean ± SD (n = 3).

To further investigate the analytical performance of SPG‐MpECis in fingerstick blood samples and its potential for true at‐home testing, we selected C‐reactive protein (CRP)—a clinically common biomarker in fingerstick blood testing—as the target analyte for validation. First, we conducted a preliminary validation of the SPG coating's ability to resist interference from whole blood obtained from a fingerstick. The results indicated that compared with bare electrodes, SPG coated electrodes could effectively maintain the stability of electrochemical signals (Figure 7a–c). As the blood concentration increased, the rate and amplitude of current signal decrease of the bare electrode were more than three times that of the SPG electrode, indicating that SPG‐MpECis has the potential to directly detect target biomarkers in fingertip blood. On this basis, we established a standard curve for CRP detection (Figure S22) and collected fingertip blood samples from 17 patients, including 6 healthy controls and 11 clinically diagnosed fever patients. The above samples were tested using SPG‐MpECis, and the results showed significant consistencybetween this method and the clinical gold standard—the immunoturbidimetric assay (Figure 7d,e). The correlation coefficient R^2^ was 0.97, and Bland‐Altman analysis showed a mean difference of 4.13. Furthermore, SPG‐MpECis system demonstrated excellent diagnostic performance for the patient population (Figure 7f,g), with an area under the ROC curve (AUC) of 1.00 and a confusion matrix indicating 100% specificity and 100% sensitivity. The complete test results for both methods are shown in Figure 7h,i. In summary, the SPG‐MpECis device developed by our research could assist in determining the infection or inflammatory status of fever patients by directly detecting CRP in fingertip blood under non central laboratory conditions.

## 3 Conclusions

In summary, we have developed a calcium‑ion‑responsive, rapid‐forming SPG hydrogel that can be coated onto electrodes within 1 min, effectively resisting nonspecific adsorption from complex biological fluids such as blood, while the incorporated graphene greatly reduces the coating resistance. Furthermore, by combining a 3D‑printed pen‐cap‐shaped magnetic guidance apparatus with a portable USB electrochemical detection module, we assembled the SPG‑MpECis platform for home‑based blood testing. The platform integrates homogeneous incubation, magnetic enrichment, and electrochemical readout into a unified workflow, shortening the overall immunoassay time to 25 min. Moreover, time‐domain averaging of the enzyme‑generated electrochemical signals reduced the coefficient of variation (CoV) to 3.4%. The SPG‐MpECis platform has been successfully applied to the direct detection of biomarkers in serum samples from 59 patients with acute myocardial infarction (AMI) and 47 patients with non‐small cell lung cancer (NSCLC), as well as in 17 fingerstick blood samples, yielding results that correlated well with reference methods, with AUC values of 0.99, 0.93 and 1.00, respectively. Collectively, this platform overcomes key barriers that hinder the practical translation of existing electrochemical POCT technologies, opening a new avenue for home‑based testing of aging populations and patients with chronic diseases.

However, in order to truly promote the application of this platform in home detection scenarios, there are still several issues that need to be addressed through further research. Firstly, the management of critical detection conditions and the collection of special samples still rely on training or professional equipment assistance, and untrained non‐professionals are at risk of operational failure, which directly affects the autonomous use of non‐professionals in home environments; Secondly, the long‐term storage performance of the entire detection kit still needs further exploration to optimize storage conditions and effective storage time, ensuring the reliability and stability of the reagents under home conditions.

## 4 Experimental Section

### 4.1 Ethics Statement

All human clinical sample collection and testing procedures in this study were conducted in strict compliance with the Declaration of Helsinki. This study was approved by the Medical Research Ethics Review Committee of the First Affiliated Hospital of Chongqing Medical University (Approval No. 2025‐844‐01). The ethics committee granted a waiver of informed consent as the samples were anonymized and the study posed minimal risk to participants. Clinical blood samples from patients with acute myocardial infarction (AMI), non‐small cell lung cancer (NSCLC), C‐reactive protein (CRP)‐evaluated inflammatory conditions, and healthy donors (HDs) were collected from the First Affiliated Hospital of Chongqing Medical University, with approvals from the medical ethics committees of the hospital. All relevant ethical regulations were followed.

### 4.2 Materials

Horseradish peroxidase (HRP) and sodium alginate (SA) were purchased from Aladdin. Anti‐CD63, Anti‐PD‐L1, and anti‐Cardiac Troponin I (cTnI) were purchased from ABclonal (Wuhan, China). The cTnI polyclonal antibody and CRP Monoclonal antibody were purchased from Proteintech (Wuhan, China). The Biotin Conjugated Human CRP Rabbit mAb was purchased from HUABIO (Zhejiang, China). Cy5‐labeled bovine serum albumin (Cy5‐BSA) was purchased from G‐GLONE (Beijing, China). Aminated polyethylene glycol (PEG‐NH_2_, 10000 Da) and and nano‐graphene powder (electrical conductivity: ≥1600 S/cm) were purchased from Macklin (Shanghai, China). Streptavidin magnetic beads were purchased from Beyotime. Calcium chloride (CaCl_2_) and Tween‐20 were purchased from Sangon Biotech. BSA, chlorogoldic acid (HAuCl_4_), polyvinylpyrrolidone K30 (PVP‐K30), L‐ascorbic acid, platinum tetrachloride (H_2_PtCl_4_), and hydrogen peroxide (H_2_O_2_) were purchased from Sigma‐Aldrich. All chemical reagents were of analytical grade purity. Solutions were prepared using deionized water (DI water) with an electrical resistivity of ≥18.2 MΩ throughout the experiment.

### 4.3 Apparatus

A portable electrochemical device was integrated with a U‐type electrochemical workstation (Shenzhen Refresh Biosensing Technology Co., Ltd., China) and a Bluetooth‐connected smartphone with a testing APP. Cyclic voltammetry (CV) and electrochemical impedance spectroscopy (EIS) measurements were performed using a CHI660E electrochemical workstation (CH Instruments, Shanghai). The working, reference, and counter electrodes of the screen‐printed carbon electrode (SPE) were carbon, Ag/AgCl, and carbon electrodes, respectively. Characteristic absorption spectra of the nanomaterials were analyzed using an ultraviolet‐visible (UV‐vis) spectrophotometer (Shimadzu Corporation, Japan), while morphological characterization was performed by transmission electron microscopy (TEM) (Hitachi, Ltd., Japan). The SPG coating and its microstructure on the electrode surface were characterized using scanning electron microscopy (SEM) (Hitachi, Japan) and atomic force microscopy (AFM) (Bruker AXS, Germany). Fluorescence imaging was acquired using a TCSSP8 confocal laser scanning microscope (CLSM, Leica Microsystems, Germany). Particle size was measured by dynamic light scattering (DLS). Contact angle values were measured using a water contact angle analyzer (KRüSS DSA100, Germany). The force‐distance curves were obtained using a Dimension Icon (Bruker, Germany), and the normal spring constant was approximately 5.00 N/m.

### 4.4 Preparation and Characterization of SPG Hydrogels and Coatings

To prepare the SPG composite hydrogel precursor solution, a 2 mL solution of SA (1.5 wt%) was thoroughly mixed with 0.04 g PEG‐NH_2_ and 0.2 wt% nano‐graphene powder under magnetic stirring and probe ultrasonication to ensure uniform dispersion. The SPG hydrogel was formed by adding a 3% CaCl_2_ solution to the SPG solution and incubating at room temperature for 1 min. The SPG coating was obtained by dispensing the SPG solution onto the substrate and then immersing it in a 3% CaCl_2_ solution for 1 min. SEM observations, energy dispersive spectroscopy (EDS) analysis, XPS, and XRD were performed on freeze‐dried S, SP, and SPG hydrogels. Surface morphology and roughness of the SPG coating were characterized using AFM, and force‐distance curve tests were conducted on S, SP, and SPG coatings. Young's modulus was calculated using NanoScope Analysis software. Furthermore, AFM was employed to characterize the edge morphology of each coating in fresh and dried states, enabling the calculation of coating thickness. Additionally, conductive atomic force microscopy (C‐AFM) was used to characterize the electrical properties of the SPG coating, with current density distribution analyzed via NanoScope Analysis.

### 4.5 Performance Characterization of SPG‐Modified Electrodes

The surface morphology of bare electrodes and SPG‐modified electrodes was characterized using SEM. Bare electrodes and SPG‐modified electrodes were tested using the CV method at scan rates ranging from 20 to 100 mV/s. Their surface redox behavior was analyzed to evaluate their electrochemical performance. CLSM was used to directly observe the adsorption of Cy5‐labeled BSA on the surfaces of both bare electrodes and SPG‐modified electrodes. The antifouling performance of the SPG‐modified electrode was evaluated using bovine serum albumin (BSA) and horseradish peroxidase (HRP) as model fouling proteins. For the HRP model, bare electrodes and SPG modified electrodes were incubated in a 1% HRP solution for 1 h, then transferred to 0.01 M PBS buffer (pH 7.4) containing hydrogen peroxide. The antifouling performance of each electrode was detected and compared using DPV. For the BSA model, bare electrodes and SPG modified electrodes were immersed in ultrapure water or 1% BSA solution for 1 h of incubation. They were then placed in PBS (0.01 M, pH 7.4) containing 5 mM K_3_ [Fe (CN)_6_]/K_4_ [Fe (CN)_6_] and 0.1 M KCl. pH 7.4) for DPV recording of current responses. The inhibition rate was calculated using the formula “*signal inhibition rate = (I_0_ − I_1_)/I_0_*,” where I_0_ and I_1_ represent the current values before and after incubation with contaminants, respectively. Anti‐BSA tests were conducted by adjusting the concentration of SA, PEG, and graphene to determine the optimal composition ratio. Subsequently, the bare electrode and SPG‐modified electrode were immersed in urine or serum solutions of varying concentrations for 1 h. The actual protein adsorption on the electrode surface was assessed by evaluating changes in DPV current, thereby evaluating the signal stability of bare electrodes and SPG‐modified electrodes in complex environments.

### 4.6 Synthesis and Characterization of MB‐Ab1 and AuPt‐Ab2

To prepare 13 nm AuNPs via the sodium citrate reduction method, a 100 mL aliquot of 1 mM chloroauric acid (HAuCl_4_) solution was heated to boiling and maintained for 5 min. Subsequently,10 mL of 38.8 mM sodium citrate solution was rapidly added to the boiling solution, and the reaction was continued for 15–20 min. Heating was terminated when the solution turned deep red. Stirring was continued until the solution cooled naturally to room temperature, yielding AuNPs with a particle size of approximately 13 nm. The resulting AuNPs were stored at 4°C for later use.

For the preparation of AuPt nanoparticles, a 155 µL portion of the above AuNPs was mixed with 100 µL of 200 g/L PVP‐K30, dilute to 5 mL with deionized water, and stir magnetically at room temperature for 5 min. Subsequently, 200 µL of 100 mg/mL L‐ascorbic acid (L‐AA) and 1000 µL of 20 mM platinum (IV) chloride (H_2_PtCl_4_) were added. The mixture was immediately placed in a 65°C oil bath and stir for 30 min. Heating was stopped when the solution color changed from red to black, and the mixture was allowed to cool to room temperature. The reaction mixture was centrifuged at 1250 × g for 12 min, washed twice with deionized water, and the resulting AuPt nanoparticles were resuspended in 2 mL of deionized water for later use.

To synthesize the AuPt‐Ab2 complex, a 200 µL of the AuPt suspension and 4 µL of 450 mg/mL antibody (Ab2) to 800 µL of PBS. The mixture was stirred at 4°C for 12 h. The product was collected by centrifugation and washed twice with PBS to remove unbound free antibody, yielding the AuPt‐Ab2 complex dispersed in PBS. Subsequently, 200 µL of 1% BSA was added and incubated at 4°C under stirring for 1 h to block non‐specific binding sites. Finally, the complex was washed with PBS and resuspended for subsequent use.

MB‐Ab1 was constructed using the biotin‐streptavidin system: a 20 µL aliquot of 10 mg/mL MB was subjected to magnetic separation, washed three times with phosphate‐buffered saline (PBS, pH 7.4) containing 0.05% Tween‐20, and resuspended in 200 µL PBS for later use. To conjugate the antibody, a 200 µL volume of 1 mg/mL MB and 4 µL of 2 mg/mL Ab1 were added to 800 µL of PBS, followed by incubation at 4°C with stirring for 8 h. The product was collected via magnetic separation, washed twice with PBS to remove unbound free antibody, and MB‐Ab1 was obtained dispersed in PBS. Subsequently, 200 µL of 1% BSA was added and incubated at 4°C with stirring for 1 h for blocking. The MB‐Ab1 was then isolated by magnetic separation, washed with PBS, and resuspended for later use. The morphological characteristics, particle size distribution, and elemental composition of MB‐Ab1 and AuPt‐Ab2 were characterized using UV‐vis, TEM, and DLS.

### 4.7 Detection Protocol of SPG‐MpECis

SPG‐MpECis: A MB‐Ab1 dispersion was mixed with a sample containing the target, followed by the addition of AuPt‐Ab2 and incubation at 37°C to form a sandwich immunocomplex (MB‐target‐AuPt). The SPG‐modified screen‐printed electrode was immersed in the reaction solution. The complex was magnetically immobilized using a magnet embedded in a 3D printing pen cap assembly. Subsequently, the electrode was immersed in PBS buffer (100 mM, pH 7.4) containing 1.5 mM H_2_O_2_ for amperometric testing. The potential was set to 800 mV, and the potential was applied for 3 min.

### 4.8 Statistical Analysis

GraphPad Prism 9.0, OriginPro 2021, ImageJ, and NanoScope Analysis were used for data preprocessing, signal quantification, and statistical evaluation. For electrochemical current readings, time‐domain signal averaging was performed during the steady‐state catalytic plateau (120–180 s) to minimize transient signal fluctuations. Outliers were evaluated using Grubbs' test (α = 0.05) and no significant outliers were detected in the current datasets. Normality of the data was assessed using the Shapiro–Wilk test (p > 0.05 considered normal), and homogeneity of variances was verified using Levene's test before statistical comparisons. The signal suppression rate was calculated using the formula (I_0_ − I_1_)/I_0_. All laboratory experiments were repeated at least three times (n = 3), and experimental data are presented as mean ± standard deviation (SD).

Statistical comparisons between two independent groups were performed using an unpaired, two‐tailed Student's t‐test. Statistical significance was denoted as *p < 0.05, **p < 0.01, ***p < 0.001, ****p < 0.0001; no significant difference was denoted as n.s. (p > 0.05). For clinical diagnostic evaluation, the sample sizes (n) for different cohorts were specified as follows: serum cTnI samples (*n* = 59; 39 samples from AMI patients, 20 healthy samples), serum EVs samples (*n* = 47; 30 samples from NSCLC patients, 17 healthy samples), and fingerstick whole blood C‐reactive protein (CRP) samples (*n* = 17; 11 samples from patients with fever, 6 healthy samples). Methodological agreement between SPG‐MpECis and clinical reference methods (CLIA and turbidimetric assay) was quantitatively assessed using linear regression (R^2^) and Bland‐Altman plots. Diagnostic sensitivity, specificity, and predictive accuracy were evaluated using receiver operating characteristic (ROC) curve analysis (quantified by the area under the curve, AUC) and confusion matrices.

## Author Contributions

H.W., S.D., and W.C. conceived and designed the project. M.L., K.S., R.H., and K.G. carried out the experiments. M.L., K.S., R.H., Y.L., L.Z., N.F., and Y.C. analyzed the data and interpreted the results. The manuscript was written through the contributions of all authors. All authors have given approval to the final version of the manuscript.

## Conflicts of Interest

The authors declare no conflicts of interest.

## Supporting information




**Supporting File**: advs77696‐sup‐0001‐SuppMat.docx.

## Data Availability

The data that support the findings of this study are available from the corresponding author upon reasonable request.
