# Supplementary material for: Rapid‐Forming Conductive Antifouling Hydrogel Coating Enables Magnetically Guided Electrochemical POCT for Direct at‑Home Blood Testing
Source: Adv Sci (Weinh). 2026 Sep 16:e77696. Online ahead of print. doi: 10.1002/advs.77696 (PMC13583235; doi:10.1002/advs.77696)
Supplement: Supplementary file 1 [file ADVS-9999-e77696-s001.docx]

Supplementary Information for

**Rapid-Forming Conductive Antifouling Hydrogel Coating Enables Magnetically Guided Electrochemical POCT for Direct At‑Home Blood Testing**

Menghan Li^1,2,#^ , Kai Su^2,#^ , Ruiwei Hu^1,#^ , Ke Gao^2^, Yuanjie Liu^3^, Lu Zhang^2^, Ningke Fan^2^, Yirong Chen^2^, Wei Cheng^1^*, Shijia Ding^2^*, Haiping Wu^1,4^*

*^1^* The Center for Clinical Molecular Medical Detection, The First Affiliated Hospital of Chongqing Medical University, Chongqing, P.R. China

*^2^* Key Laboratory of Clinical Laboratory Diagnostics (Ministry of Education), College of Laboratory Medicine, Chongqing Medical University, Chongqing, China

*^3^* Department of Clinical Laboratory, Qianjiang Key Laboratory of Chongqing Qianjiang Central Hospital Laboratory medicine Chongqing University Qianjiang Hospital (Qianjiang Central Hospital of Chongqing) , Chongqing, China

*^4^* Sichuan-Chongqing Joint Key Laboratory for Pathology and Laboratory Medicine

Jinfeng Laboratory, Chongqing, P. R. China

*Correspondence: Shijia Ding ([dingshijia@163.com](mailto:cqmuwhp@163.com)), Wei Cheng (chengwei@ hospital.cqmu.edu.cn) and Haiping Wu (cqmuwhp@163.com)

^#^ These authors contributed equally to this work.

**Keywords:** Electrochemical POCT; Anti-fouling hydrogel coating; Magnetic-guided enrichment; Immunoassay; At-home testing

**This file includes:**

Figure S1 to S22

Table S1 to S2

**Figure S1.** SEM and TEM Characterization of Graphene.

**Figure S2.** Electrochemical performance comparison of SPG hydrogel coatings prepared with aminated PEG (NH_2_-PEG-NH_2_) versus standard PEG

**Figure S3.** Image of the rapid formation of SPG hydrogel.

**Figure S4.** Scanning electron microscopy characterization of S and SP hydrogels.

| **Element** | **Line Type** | **Weight %** | **Weight % Sigma** | **Atomic %** |
| --- | --- | --- | --- | --- |
| **C** | **K series** | **49.61** | **0.94** | **63.07** |
| **O** | **K series** | **27.57** | **0.72** | **26.32** |
| **Na** | **K series** | **4.47** | **0.17** | **2.97** |
| **Cl** | **K series** | **13.30** | **0.32** | **5.73** |
| **Ca** | **K series** | **5.06** | **0.22** | **1.93** |
| **Total** |  | **100.00** |  | **100.00** |

**Figure S5.** EDS elemental mapping and quantitative analysis results for SPG hydrogels.

**Figure S6.** (a) XPS survey spectra, (b) XRD crystalline, and (c) Fourier-transform infrared spectroscopic characterization of S, SP, and SPG hydrogels.

**Figure S7.** SEM characterization of S, SP, and SPG modified electrodes.

**Figure S8.** Analysis of the (a) physical thickness of SPG hydrogel coatings prepared using different volumes of precursor solutions and (b, c) optimization of interfacial electrochemistry.

**Figure S9.** AFM topography images of the S and SP hydrogel interfaces and the corresponding C-AFM current images.

**Figure S10.** Force-distance curve results for S, SP, and SPG hydrogels against simulated contaminants.

**Figure S11.** AFM measurements were conducted to determine changes in the thickness of S, SP, and SPG hydrogel coatings under dry and wet conditions.

**Figure S12.** SEM morphology and quantitative surface gray-value analysis evaluating the antifouling performance of (a) bare electrodes and (b) SPG modified electrodes before and after 1 h serum incubation.

**Figure S13.** TEM characterization of MB, and UV absorption characterization at 280 nm of MB and MB-Ab1.

**Figure S14.** TEM and DLS characterization of Au and AuPt; elemental mapping of AuPt; UV absorption characterization of AuPt and AuPt-Ab2 at 280 nm.

**Figure S15.** (a)Schematic diagrams of the construction of MB-Ab1 and AuPt-Ab2. (b,c) TMB colorimetric assays and UV absorption spectra confirm the formation of immune complexes in the presence and absence of the target, as well as the feasibility of the detection system.

**Figure S16.** Design drawing for a 3D-printed Magnetic Pen Cap Mold.

**Figure S17.** Conditions were optimized for (a) the C_MB_/C_Ab1_ ratio, (b) AuPt-Ab2 concentration, and (c) reaction time in the assay system.

**Figure S18.** Segmental data analysis of the electrical signals obtained from measuring different concentrations of cTnI at 10 s intervals within the three time windows of (a-b) 0-60 s, (c-d) 60-120 s, and (e-f) 120-180 s.

**Figure S19.** Analyze the (a) specificity, (b) stability, and (c-d) reproducibility of the cTnI electrical signals measured at 10-second intervals within the three time windows of 0-60 s, 60-120 s, and 120-180 s.

**Figure S20.** Analyze the (a-c) linearity, (d) specificity, (e) stability, and (f-g) reproducibility of the EVs electrical signals measured at 10-second intervals within the three time windows of 0-60 s, 60-120 s, and 120-180 s.

**Figure S21.** Segmental data analysis of the electrical signals obtained from measuring different concentrations of EVs at 10 s intervals within the three time windows of (a-b) 0–60 s, (c-d) 60–120 s, and (e-f) 120–180 s.

**Figure S22.** linearity of the CRP electrical signals measured at 10 s intervals within the time window of 120-180 s. Data are presented as mean ± SD (n = 3).

**Table S1.** A Comparison of SPG Coating Strategies with Various Sensor Interface Preparation Methods Reported in the Literature.

| Coating method | Modification steps | Time of modification | Ref |
| --- | --- | --- | --- |
| SAM by Au-S bond | 1 | 12 h | (1) |
| SAM by Au-S bond | 1 | 18 h | (2) |
| SAM by Si-O bond | 1 | 12 h | (3) |
| LBL by charge attraction | 2 | 4 h | (4) |
| LBL by charge attraction | 7 | 6 h | (5) |
| LBL by hydrophobic interactions | 3 | 15 h | (6) |
| PDA functionalized coating by ‘graft to’ | 1 | 24 h | (7) |
| PDA functionalized coating by co-deposition | 1 | 18 h | (8) |
| PDA functionalized coating by biomimetic adhesion | 2 | 36 h | (9) |
| Hydrogel coating by ionic crosslinking | 1 | 1 min | This work |

**Table S2.** Detailed specifications of the antibodies used in the sandwich immunoassays.

| **Function** | **Antibody Name** | **Cat. No.** | **Host / Type** | **Reported Sensitivity / Affinity Parameters** |
| --- | --- | --- | --- | --- |
| cTnI Capture | Biotinylated Rabbit anti-Human cTnI mAb | RMK0301 | Rabbit / Monoclonal | Recommended Conc.: 0.03–0.1 μg/mL; |
| cTnI Detection | Cardiac Troponin I Polyclonal antibody | 21652-1-AP | Rabbit / Polyclonal | High Sensitivity: WB (1:80000); IHC: 1:400–1:1000; |
| EVs Capture | Biotin Anti-CD63 antibody | ab134331 | Mouse / Monoclonal | Recommended Conc.: 0.6 μg/mL for Flow Cytometry; |
| EVs Detection | PD-L1/CD274 Rabbit mAb | A19135 | Rabbit / Monoclonal | Recommended Conc.: 1 μg/mL for ELISA; WB: 1:1000–1:2000 |
| CRP  Capture | Biotin Conjugated Human CRP Rabbit mAb | HA722616B | Rabbit / Monoclonal | Detection range: 617–50,000 pg/mL |
| CRP Detection | Human C-Reactive Protein/CRP mAb | 66250-1-Ig | Mouse / Monoclonal | Recommended Conc.:  WB: 1:2000–1:10000 |

**References**

1.	Zhang Z, Wang Y, Mei Z, et al., “Incorporating Hydrophobic Moieties into Self-Assembled Monolayers to Enable Electrochemical Aptamer-Based Sensors Deployed Directly in a Complex Matrix.” *ACS Sensors*. 2022;7(9):2615-2624. doi:10.1021/acssensors.2c00995

2.	Inkpen MS, Liu Z, Li H, Campos LM, Neaton JB, Venkataraman L., “Non-chemisorbed gold–sulfur binding prevails in self-assembled monolayers.” *Nature Chemistry*. 2019;11(4):351-358. doi:10.1038/s41557-019-0216-y

3.	Singh M, Kaur N, Drera G, Casotto A, Sangaletti L, Comini E., “SAM Functionalized ZnO Nanowires for Selective Acetone Detection: Optimized Surface Specific Interaction Using APTMS and GLYMO Monolayers.” *Advanced Functional Materials*. 2020;30(38):2003217. doi:10.1002/adfm.202003217

4.	Mariani S, Robbiano V, Strambini LM, et al., “Layer-by-layer biofunctionalization of nanostructured porous silicon for high-sensitivity and high-selectivity label-free affinity biosensing.” *Nature Communications*. 2018;9(1):5256. doi:10.1038/s41467-018-07723-8

5.	Jashni E, Hosseini SM, Shabanian M, Sadrzadeh M., “Silane functionalized graphene oxide-bound polyelectrolyte layers for producing monovalent cation permselective membranes.” *Separation and Purification Technology*. 2021;278:119583. doi:10.1016/j.seppur.2021.119583

6.	Huang Y, Wu H, Xie N, et al., “Conductive Antifouling Sensing Coating: A Bionic Design Inspired by Natural Cell Membrane.” *Advanced Healthcare Materials*. 2023;12(13):2202790. doi:10.1002/adhm.202202790

7.	Li G, Cheng G, Xue H, Chen S, Zhang F, Jiang S., “Ultra low fouling zwitterionic polymers with a biomimetic adhesive group.” *Biomaterials*. 2008;29(35):4592-4597. doi:10.1016/j.biomaterials.2008.08.021

8.	Ren PF, Yang HC, Jin YN, Liang HQ, Wan LS, Xu ZK., “Underwater superoleophobic meshes fabricated by poly(sulfobetaine)/polydopamine co-deposition.” *RSC Advances*. 2015;5(59):47592-47598. doi:10.1039/C5RA05937B

9.	Shevate R, Kumar M, Karunakaran M, Hedhili MN, Peinemann KV., “Polydopamine/Cysteine surface modified isoporous membranes with self-cleaning properties.” *Journal of Membrane Science*. 2017;529:185-194. doi:10.1016/j.memsci.2017.01.058
